# Epileptogenic focus localization: a new approach

**DOI:** 10.1186/2197-7364-2-S1-A81

**Published:** 2015-05-18

**Authors:** Vânia Tavares, André Santos Ribeiro, Carlos Capela, Luís Cerqueira, Hugo Alexandre Ferreira

**Affiliations:** Institute of Biophysics and Biomedical Engineering, Faculty of Sciences of the University of Lisbon, Lisboa, Portugal; Centre for Neuropsychopharmacology, Division of Brain Sciences, Department of Medicine, Imperial College London, London, UK; Department of Neuroradiology, Centro Hospitalar Lisboa Central, Lisbon, Portugal

Epilepsy is one of the most important chronic neurological disorders worldwide affecting more than 50 million people of all ages. Among these, almost 20% of epilepsy cases are uncontrollable and have an unknown source of this abnormal electrical activity. Present techniques for the detection of epileptogenic foci include electroencephalography (EEG), positron emission tomography, and multimodal EEG/functional magnetic resonance imaging (fMRI), all with limitations in terms of spatial and temporal resolutions. In order to overcome some of those limitations a novel approach using fMRI alone was developed based on the hypotheses that the epileptogenic focus shows Blood Oxygen Level Dependent (BOLD) temporal profiles distinct from the remaining brain parenchyma during interictal activity and that the epileptogenic focus BOLD signals show lower complexity than healthy parenchyma. In this novel approach, bi-dimensional temporal clustering analysis, a data-driven technique, was used to identify brain regions with similar temporal profiles. Then, the BOLD signals of these regions were assessed regarding complexity using detrended fluctuation analysis and also using a modified multiscale entropy algorithm in order to identify which of those regions corresponded to epileptogenic tissue. In order to demonstrate the applicability of the developed method three epileptic patients were analyzed comprising two types of epilepsy: unilateral and bilateral temporal lobe epilepsies. The results showed that this method is able to detect the brain regions associated with epileptogenic tissue. The results also showed that the epileptogenic focus influences the dynamics of related brain networks. This could be a key factor in the applicability of this method to other epilepsy cases. Finally, new perspectives are envisioned concerning the use of this method in the medical care of epilepsy. In particular, by improving this method using simultaneous structural, functional, and metabolic information with hybrid MRI-PET scanners for the validation of epileptogenic focus.

